# Alpha‐kinase1 promotes tubular injury and interstitial inflammation in diabetic nephropathy by canonical pyroptosis pathway

**DOI:** 10.1186/s40659-023-00416-7

**Published:** 2023-02-02

**Authors:** Xinyuan Cui, Yifu Li, Shuguang Yuan, Yao Huang, Xiaojun Chen, Yachun Han, Zhiwen Liu, Zheng Li, Yang Xiao, Youliang Wang, Lin Sun, Hong Liu, Xuejing Zhu

**Affiliations:** 1 Hunan Key Laboratory of Kidney Disease and Blood Purification, Department of Nephrology, The Second Xiangya Hospital of Central South University, Changsha, 410011 Hunan China; 2 Center for Medical Research, The Second Xiangya Hospital of Central South University, Changsha, China; 3 Key Laboratory of Diabetes Immunology, Department of Metabolism and Endocrinology, National Clinical Research Center for Metabolic Diseases, Ministry of Education, The Second Xiangya Hospital of Central South University, Changsha, China

**Keywords:** Diabetic nephropathy, Alpha‐kinase 1, Pyroptosis, Tubular injury

## Abstract

**Background:**

Alpha‐kinase 1 (ALPK1) is a master regulator in inflammation and has been proved to promote renal fibrosis by promoting the production of IL-1β in diabetic nephropathy (DN) mice. Pyroptosis is involved in high glucose (HG)-induced tubular cells injury, characterized by activation of Gasdermin D (GSDMD) and the release of IL-1β and IL-18, resulting in inflammatory injury in DN. It is reasonable to assume that ALPK1 is involved in pyroptosis-related tubular injury in DN. However, the mechanism remains poorly defined.

**Methods:**

Immunohistochemistry (IHC) staining was performed to detect the expression of pyroptosis- and fibrosis-related proteins in renal sections of DN patients and DN mice. DN models were induced through injection of streptozotocin combined with a high-fat diet. Protein levels of ALPK1, NF-κB, Caspase-1, GSDMD, IL-1β, IL-18 and α-SMA were detected by Western blot. HK-2 cells treated with high-glucose (HG) served as an in vitro model. ALPK1 small interfering RNA (siRNA) was transfected into HK-2 cells to down-regulate ALPK1. The pyroptosis rates were determined by flow cytometry. The concentrations of IL-1β and IL-18 were evaluated by ELISA kits. Immunofluorescence staining was used to observe translocation of NF-κB and GSDMD.

**Results:**

The heat map of differentially expressed genes showed that ALPK1, Caspase-1 and GSDMD were upregulated in the DN group. The expression levels of ALPK1, Caspase-1, GSDMD and CD68 were increased in renal biopsy tissues of DN patients by IHC. ALPK1expression and CD68^+^ macrophages were positively correlated with tubular injury in DN patients. Western blot analysis showed increased expressions of ALPK1, phospho-NF-κB P65, GSDMD-NT, and IL-1β in renal tissues of DN mice and HK-2 cells, accompanied with increased renal fibrosis-related proteins (FN, α-SMA) and macrophages infiltration in interstitial areas. Inhibition of ALPK1 attenuated HG-induced upregulation expressions of NF-κB, pyroptosis-related proteins Caspase-1, GSDMD-NT, IL-1β, IL-18, α-SMA, and pyroptosis level in HK-2 cells. Also, the intensity and nuclear translocation of NF-κB and membranous translocation of GSDMD were ameliorated in HG-treated HK-2 cells after treatment with ALPK1 siRNA.

**Conclusions:**

Our data suggest that ALPK1/NF-κB pathway initiated canonical caspase-1-GSDMD pyroptosis pathway, resulting in tubular injury and interstitial inflammation of DN.

**Supplementary Information:**

The online version contains supplementary material available at 10.1186/s40659-023-00416-7.

## Background

Diabetic nephropathy (DN) is one of the most common and severe complications of diabetes mellitus (DM). The prevalence of DN has increased dramatically over the past decade, especially in developing countries. Exploring the pathogenic mechanisms is critical in developing new strategies for the treatment of DN. It has been generally recognized that immune and inflammatory responses play an important role in the development and progression of DN, especially on tubular injury and renal fibrosis [1, 2]. However, molecular mechanism is still not fully understood.

Pyroptosis is an immunogenic form of programmed cell death. It involves cell swelling and lysis, causes release of pro-inflammatory factors and triggers strong inflammatory injury. Canonical pathway of pyroptosis is characterized by activation of caspase-1, which consequently cleaves gasdermin D (GSDMD). GSDMD is the important executive protein of pyroptosis, which could release the N-terminal domain after cleavage by caspase family [3, 4]. The N-terminal of GSDMD (GSDMD-NT) mediates pore formation by binding to the cell membrane, resulting in cytoplasmic efflux and release of interleukin-1β (IL-1β) and interleukin-18 (IL-18) [5]. Our previous study has confirmed that canonical pyroptotic death plays an important role in the mechanism of renal tubular epithelial cells (TECs) injury of DN [6]. However, the main regulatory proteins and mechanism of the pyroptosis injury of renal tubular cells in DN are not clear.

Alpha‐kinase 1 (ALPK1) is a cytosolic pattern recognition receptor (PRR) and belongs to the alpha‐kinase family. It has been proved that ALPK1 is a master regulator in inflammation and innate immunity [7–9]. Liu et al. have identified that ALPK1 aggravates metabolic disturbances by activating NOD-like receptor protein 3 (NLRP3) and driving IL-1β-mediated inflammatory conditions, suggesting the possible regulatory role of ALPK1 in pyroptosis [10]. Furthermore, longitudinal population-based genetic epidemiology has reported that rs2074379 (G→A) and rs2074388 (A→G) of ALPK1 gene were significantly associated with the prevalence of type 2 DM [11]. Kuo et al. have reported that ALPK1 expression is increased in human kidney‐2 cells treated with high glucose (HG). In addition, diabetic mice model with ALPK1 over-expression showed accelerated early nephropathies [12]. These let us speculate that ALPK1 might be involved in mediating pyroptosis pathway in HG-induced tubular injury of DN.

The aims of this study were to determine the effect of ALPK1 on the pyroptosis of TECs and the related molecular mechanism under high glucose environment, providing new ideas for the pathogenesis of renal tubular injury in DN patients.

## Materials and methods

### Bioinformatics analysis

The dataset GSE133598 was downloaded from NCBI’s Gene Expression Omnibus database to find differentially expressed genes (DEGs). The microarray data were analyzed and the DEGs associated with DN were screened by the GEO2R online tool [13] with |LogFC| > 1 and P ≤ 0.05. A heatmap was plotted by the Complex Heatmap package in R software version 3.6.2 for data analysis and visualization. DEGs were subjected to Gene Ontology (GO) functional analysis via DAVID online tools (https://david.ncifcrf.gov/tools.jsp). Enriched pathways in Biological Process (BP) were explored. Protein–protein interaction (PPI) network was generated through the Search Tool for the Retrieval of Interacting Genes/Proteins (STRING, https://string-db.org/) and visualized via Cytoscape software v.3.6.0 to find potential target genes of ALPK1.

### Animal experimental design

Eight-week-old male C57BL/6 mice were randomly divided into negative control group (NC) and diabetic nephropathy group (DN) (n = 3 in each group). To generate DN mouse model, mice were feed with 45% Kcal high fat diet (HFD) (No. D12451, Research Diets, Inc., New Brunswick, NJ) for 4 weeks and then administrated a single dose of STZ (100 mg/kg in 10 mM citrate buffer, pH = 4.5) intraperitoneally. Three days after the injection, the blood glucose concentration was randomly measured, with values ≥ 16.7 mmol/L for 3 times indicating successful T2D mouse model. The mice continued to be fed with HFD for 8 weeks. The body weight and blood glucose were monitored every week since STZ injection. Mice were anaesthetized and sacrificed at the end of 8 weeks of HFD feeding. The renal cortical tissues were dissected for protein extraction for subsequent analysis. Animal experiments were approved by the Animal Care and Use Committee of Second Xiangya Hospital of Central South University (2020531).

### Histological and immunohistochemical staining

Human kidney biopsy tissues of DN patients (n = 8) and glomerular minor lesion (GML) patients (n = 5) were recruited. GML is identified as minor morphological lesions by renal biopsy without DM. The percentage of tubular atrophy and interstitial fibrosis (IFTA) were assessed by two pathologists. The Ethics Committee of the Second Xiangya Hospital approved the protocol for this study. All patients signed approved informed consent forms. Renal tissues from mice were routinely processed, embedded in paraffin, sectioned (3–4 μm) and then subjected to HE staining.

Renal sections from human and mice were deparaffinized and rehydrated before being subjected to antigen retrieval in a microwave oven. Immunohistochemistry (IHC) was performed using anti-ALPK1 (1:200, Immunoway), CD68 (ZM-0060, ZSGB-Bio), F4/80 (GB11027, Servicebio), α-SMA (ab5694, abcam) and FN (ab2413, abcam) antibody as primary antibodies followed by secondary antibodies for2h. Then the slides were developed using a DAB detection kit. The human and mouse tissue sections were examined by light microscopy. Image J software (National Institutes of Health, Bethesda, MD) was used to measure the average intensity of at least 20 randomly selected fields.

### Cell culture and treatments

Human kidney proximal tubular epithelial cells (HK-2) (ATCC, USA) were cultured in DMEM/F12 (Gibco) medium supplemented with 10% fetal bovine serum and antibiotics in a humidified 5% CO_2_ incubator at 37 °C. The cells were exposed to high-glucose (HG) treatment when an approximately 80% confluence was reached and then were divided into four groups as follows: (A) 5.5 mM d-glucose (control/LG group), (B) 30 mM d-glucose (HG group), (C) 30 mM d-glucose and ALPK1 siRNA (HG + ALPK1 siRNA group), and (D) 30 mM d-glucose and negative control siRNA (HG + negative control group).

### Transfection experiments

ALPK1-specific siRNA and control siRNA were purchased from RiboBio Company (Guangzhou, China). The sequence of ALPK1 siRNA was: 5′-CGTGGCACGTGTTTATTGT-3′. HK-2 cells were transfected with ALPK1-specific siRNA (50 nmol/L) using Lipofectamine® 3000 Reagent (Invitrogen, USA) according to the manufacturer’s recommendations. Cells were treated with 30 mM d-glucose for 24 h after siRNA transfection.

### Western blotting (WB)

Total protein from HK-2 cells was lysed using RIPA lysis buffer (Beyotime, Shanghai, China). Cell lysates were centrifuged at 12,000 rpm for 15 min at 4 °C and then the supernatant was collected. BCA protein assay (Beyotime, Shanghai, China) was used to test protein concentration. 8–12% SDS-PAGE gel was prepared for separation of the samples which were transferred onto PVDF membrane (Millipore, MA, USA) by a Trans-Blot Transfer Slot (Bio-Rad, USA). The membrane was blocked with 5% skimmed milk for 2 h at room temperature and incubated with specific primary antibodies for overnight at 4 °C. The secondary antibody (anti-mouse/rabbit IgG, Proteintech, Wuhan, China) was added and then incubated for 1 h at room temperature. ECL reagent (Millipore, MA, USA) was used to detect the immunoreactive signals. Primary antibodies were used as follows: β-actin (1:3000, 20536-1-AP, Proteintech), GAPDH (1:5000, 10494-1-AP, Proteintech), Histone H3 (1:1,000, #9715, CST), ALPK1 (1:1000, YT5001, Immunoway), pro Caspase-1 + p10 + p12 (1:1000, ab179515, Abcam), GSDMD (1:1000, ab210070, Abcam), NF-κB (nuclear factor kappa B) p65 (1:1000, #8242, CST), Phospho-NF-κB p65 (1:1000, #3033, CST), mouse IL-1β (1:1000, #12242, CST), IL-18 (1:1000, 60070-1-Ig, Proteintech), α-SMA (1:1000, A5228, Sigma).

### Flow cytometry analysis of pyroptosis

Flow cytometry assay was conducted to determine the levels of cell pyroptosis using FAM-FLICA Caspase-1 Assay Kit (ICT, USA). After HG treatment, HK-2 cells were harvested and washed with phosphate buffered saline (PH = 7.4). Cells were suspended in wash buffer and then stained with FLICA and propidium iodide according to the manufacturer’s instructions. The percentage of pyroptotic cells was quantified with application of FACS calibur flow cytometer (BD Biosciences, San Jose, USA). Cells that were positive for both dyes were considered pyroptotic cells.

### Immunofluorescence (IF)

After treatments, cells were fixed with 4% paraformaldehyde for 20 min followed by permeabilization incubation with PBS containing 0.5% Triton X-100. Cells were sealed with 3% BSA for 1 h and incubated with primary antibodies anti-GSDMD (1:100, 20770-1-AP, Proteintech) and anti-NF-κB p65 (1:200, #8242, CST) in a cassette at 4 °C overnight. The next day, cells were incubated with fluorescently labeled secondary antibodies (Alexa Fluor®488; ab150077, Abcam) for 1 h at room temperature in the dark. DAPI was used to stain the nucleus for 10 min and the fluorescence signals were viewed under fluorescence microscope and the images were analyzed with Image J software.

### Nuclear cytoplasmic fractionation

The nuclear and cytoplasmic proteins were extracted and isolated according to NE-PER Nuclear and Cytoplasmic Extraction Reagents kit (Thermo Fisher Scientific) and its manufacturer’s instructions.

### Enzyme-linked immunosorbent assay

IL-18 levels in supernatant and IL-1β levels in urine and serum were detected with commercial enzyme-linked immunosorbent assay (ELISA) kits (Cusabio; Wuhan). ELISA was performed according to the instructions of the manufacturer.

### Data analysis

All statistical analyses were conducted using GraphPad Prism version 8.0 (GraphPad Software, San Diego, CA, USA). Data are presented as mean ± SD. Significant differences between multiple groups were evaluated by one-way analysis of variance, while the differences between two groups were evaluated by Student’s t-test. The correlations between two numerical variables were determined through Pearson correlation analysis. The differences were considered statistically significant at P-value < 0.05.

## Results

### Bioinformatics analysis

**Fig. 1:**
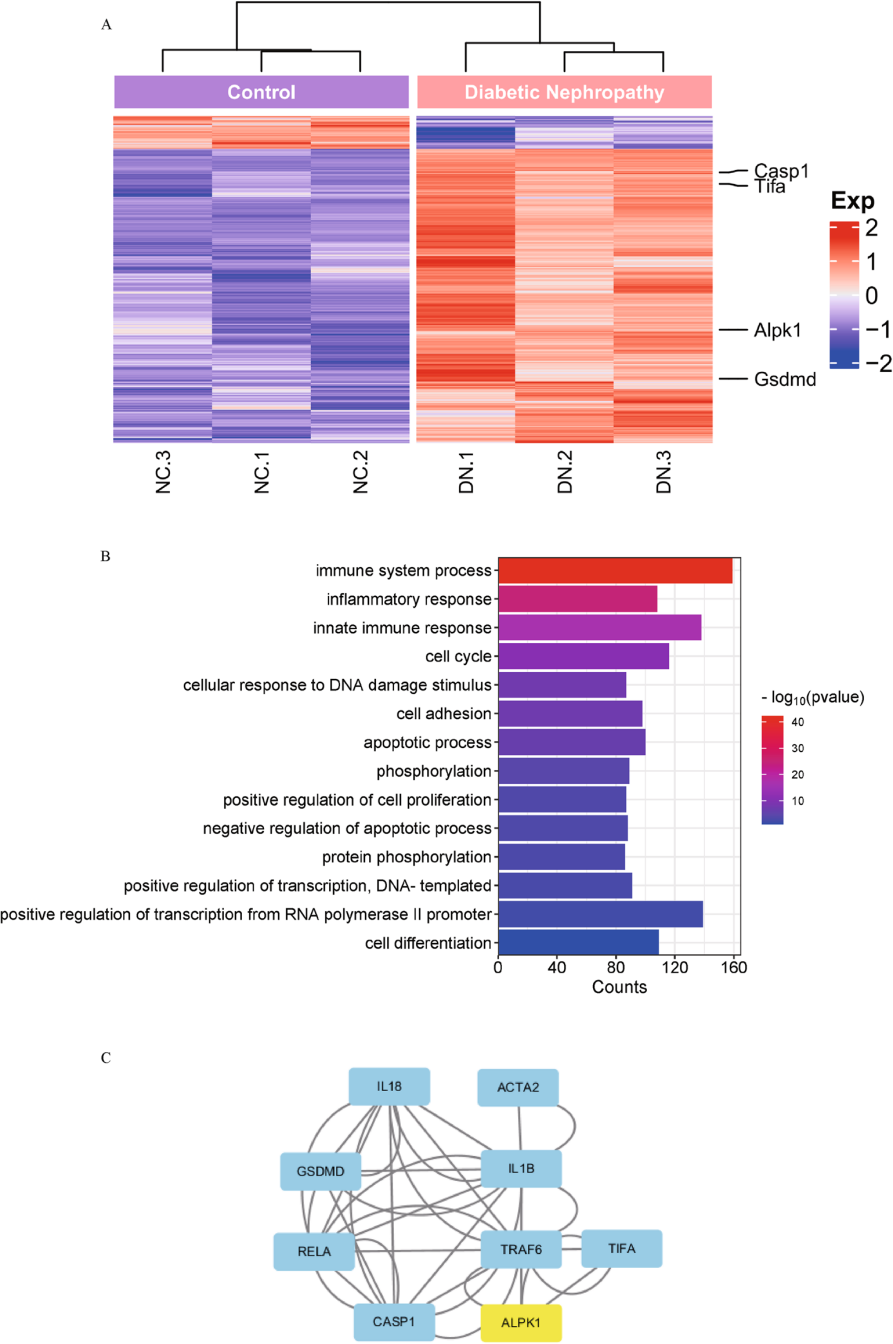
**A** Clusters of DEGs in the renal tissues of diabetic nephropathy and control mice. Each column represents a mouse sample and each row represents a gene. Red color indicates upregulation, and blue shows downregulation. Each group has 3 samples. **B** Biological process enrichment analysis of DEGs. **C** Protein–protein interaction (PPI) network of potential target genes of ALPK1. *DEGs* differentially expressed genes, *DN* diabetic nephropathy, *NC* negative control

To explore the molecular mechanism of ALPK1 involved in DN, we performed bioinformatics analysis using GEO database. Differential expression analysis of genes (DEGs) in the GSE133598 dataset [14] was performed and 2481 DEGs were found in diabetic mice compared with control mice (Fig. 1A). The heat map of DEGs showed that ALPK1, caspase-1 and GSDMD were upregulated in the DN group (Fig. 1A). Gene ontology (GO) analysis of DEGs shows the top 15 terms which have the highest enrichment scores include immune system process, innate immune response and inflammatory response, according to the biological process (BP). ALPK1 and GSDMD are co-enriched in immune system process and innate immune response pathway (Fig. 1B). PPI network using STRING database and Cytoscape software show ALPK1 associates NF-κB with pyroptosis-related proteins in the protein interaction network (Fig. 1C). The results enlighten us that ALPK1 may influence pyroptosis-related proteins through NF-κB in DN.

### ALPK1 expression and macrophages infiltration were positively correlated with tubular injury in DN patients

**Fig. 2:**
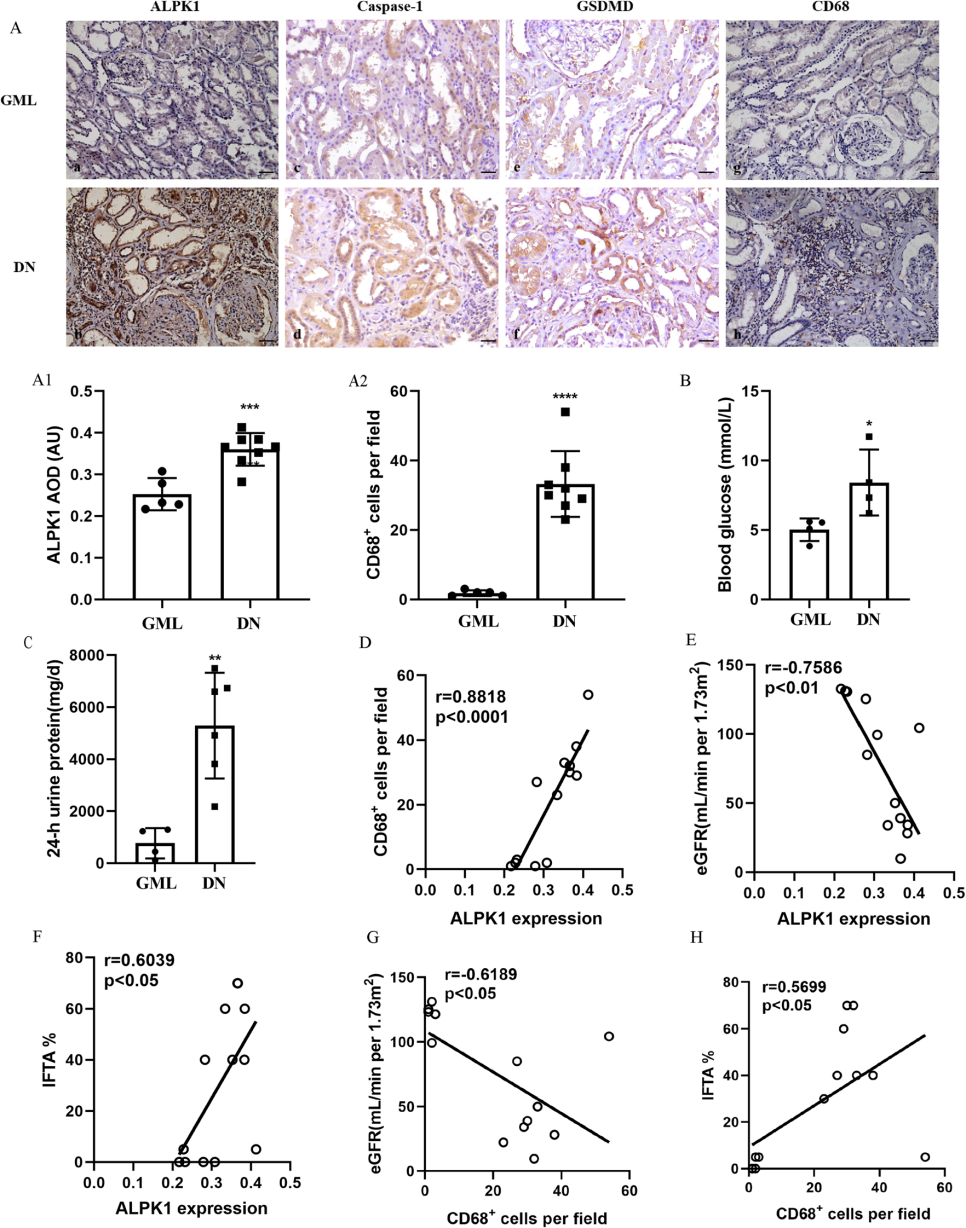
**A** IHC staining of ALPK1 (**a**, **b**), Caspase-1 (**c**, **d**), GSDMD (**e**, **f**) and CD68 (**g**, **h**) in renal tissues (scale bar, 50 μm; magnification ×200). **A1** Renal cortical relative expression of ALPK1 in renal biopsies of patients with GML and DN. **A2** Quantification of the number of CD68^+^ macrophages in renal biopsies of patients with GML and DN. Blood glucose levels (**B**) and 24-h urine protein levels (**C**) in DN and GML patients. Correlations between ALPK1 expression and the number of CD68^+^ macrophages (**D**), eGFR (**E**) and tubular atrophy (IFTA) scores (**F**). Correlations between the number of CD68^+^ macrophages per field and eGFR (**G**). Correlations between the number of CD68^+^ macrophages per field and tubular atrophy (IFTA) scores (**H**). *GML* glomerular minimal lesion, *DN* diabetic nephropathy, *AOD* Average Optical Density, *AU* arbitrary units, *eGFR* estimated glomerular filtration rate, *IFTA* interstitial fibrosis and tubular atrophy. Data were presented as mean ± SD (*P < 0.05, **P < 0.01, ***P < 0.001, ****P < 0.0001)

IHC was carried out to investigate the levels of ALPK1, caspase-1, GSDMD and CD68 in the renal tissues collected from patients with DN and glomerular minor lesion (GML) (Fig. 2A). The clinical characteristics of patients were shown in Additional file 1: Table S1. Expressions of ALPK1, caspase-1, GSDMD were elevated in tubular cells and CD68^+^ macrophages were increased in renal interstitium of DN patients. As shown in Fig. 2B, C, DN patients had higher levels of blood glucose and 24-h urine protein compared with GML patients. ALPK1 expression level was positively correlated with the number of CD68^+^ macrophages (r = 0.8818, p < 0.0001, Fig. 2D). IFTA scores reflect chronic morphological changes of tubular injury. Correlation analysis showed that ALPK1 expression was negatively correlated with eGFR (r = − 0.7586, p < 0.01, Fig. 2E) and positively correlated with IFTA scores (r = 0.6039, p < 0.05, Fig. 2F). Infiltration of CD68^+^ macrophages was also negatively correlated with eGFR (r = − 0.6189, p < 0.05, Fig. 2G) and positively correlated with IFTA scores (r = 0.5699, p < 0.05, Fig. 2H).

### Activation of ALPK1/NF-κB pathway is involved in pyroptosis-related inflammatory injury in DN mice

**Fig. 3:**
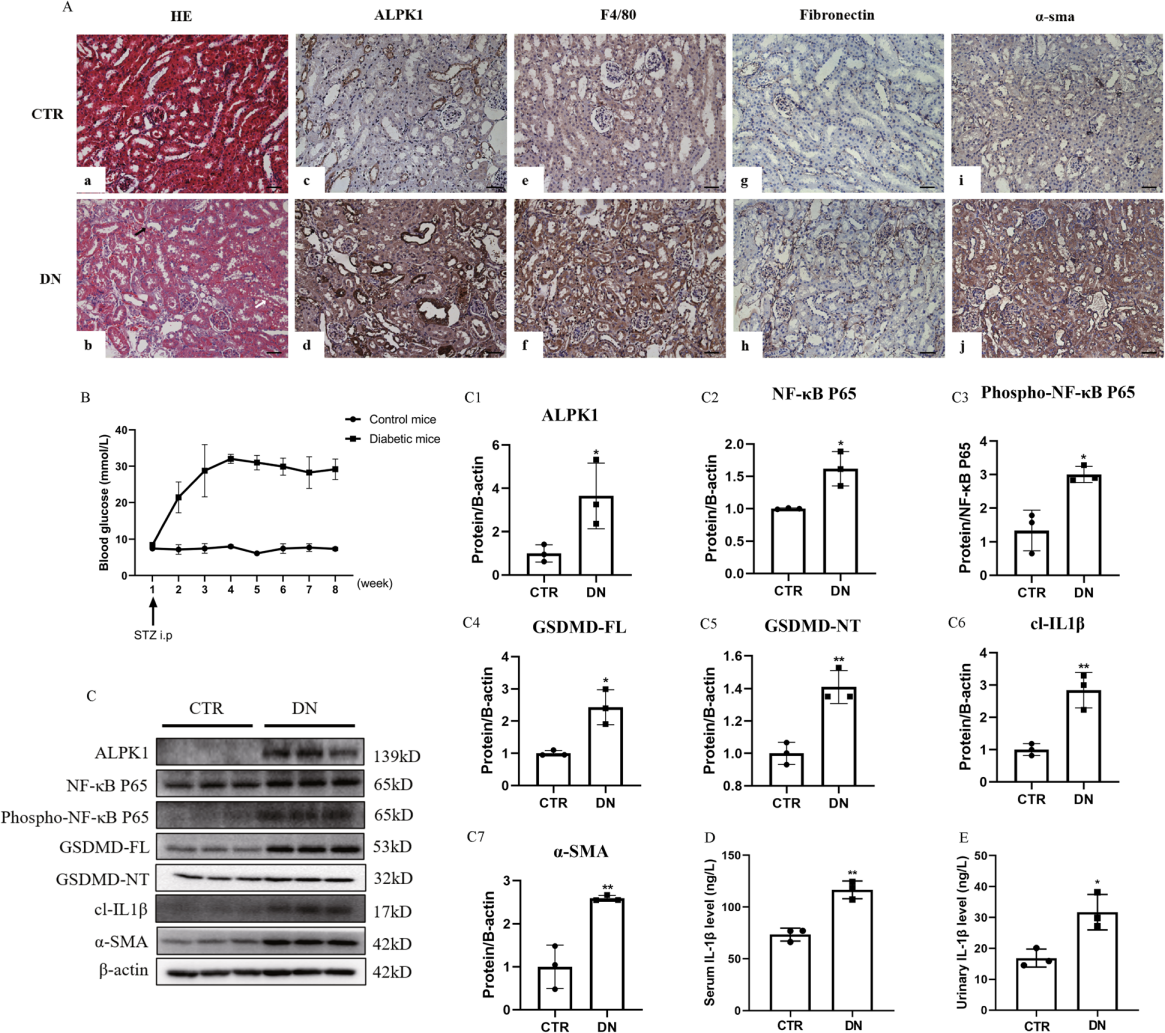
**A** Kidney sections stained with HE in control (**a**) and DN mice (**b**). Loss of brush border (black arrow shows) and vacuolar degeneration (white arrow shows) were seen. IHC staining of ALPK1 (**c**, **d**), F4/80 (**e**, **f**), fibronectin (**g**, **h**) and α-SMA (**i**, **j**) in renal tissues (scale bar, 50 μm; magnification ×200). **B** Blood glucose levels. **C** Western blotting and densitometric analysis of ALPK1 (**C1**), NF-κB P65 (**C2**), phospho-NF-κB P65 (**C3**), GSDMD-FL (**C4**), GSDMD-NT (**C5**), cl-IL-1β (**C6**) and α-SMA (**C7**) expressions in kidney tissues of control and DN mice. **D** Serum IL-1β levels. **E** Urinary IL-1β levels. *DN* diabetic nephropathy. Experiments were performed in triplicate. Data were presented as mean ± SD (*P < 0.05, **P < 0.01)

We further examined the ALPK1/NF-κB pathway and pyroptosis-related proteins in DN mice. HE staining revealed loss of brush border (Fig. 3Ab, black arrow shows) and vacuolar degeneration (Fig. 3Ab, white arrow shows) of proximal renal tubules in DN mice (Fig. 3Ab) compared with control group (Fig. 3Aa). As shown by Fig. 3A, DN mice had increased expression of ALPK1 in tubular cells (Fig. 3Ad). Expressions of fibronectin (Fig. 3Ah), α-SMA (Fig. 3Aj) and number of F4/80^+^ macrophages (Fig. 3Af) were also increased in the renal interstitium in DN mice. Blood glucose level is increased in DN mice (Fig. 3B). Western blot analysis showed increased expressions of ALPK1 (Fig. 3C, C1), NF-κB P65 (Fig. 3C, C2), phospho-NF-κB P65 (Fig. 3C, C3), GSDMD-FL (Fig. 3C, C4), GSDMD-NT (Fig. 3C, C5), cleaved IL-1β (Fig. 3C, C6) and α-SMA (Fig. 3C, C7) in renal tissues of DN mice. In addition, higher levels of serum (Fig. 3D) and urinary (Fig. 3E) IL-1β were observed in DN mice. These data suggested the presence of pyroptosis in DN mice and ALPK1/NF-κB pathway might be involved in pyroptosis-related tubular injury and interstitial inflammation of DN mice, resulting in renal fibrosis.

### ALPK1 increased in time- and concentration-dependent manner in HK-2 cells by high glucose treatment

**Fig. 4:**
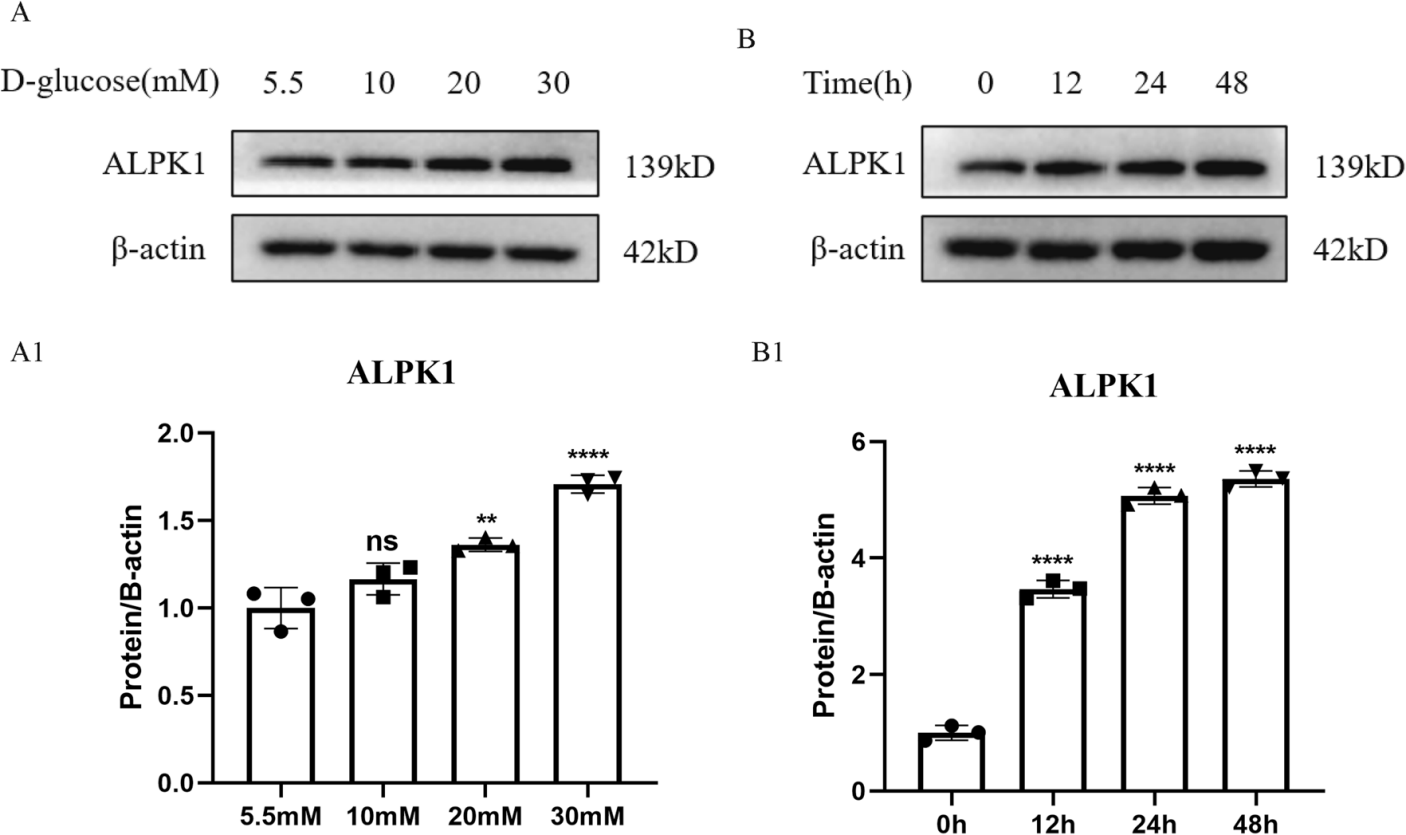
**A**, **A1** The protein levels of ALPK1 in HK-2 cells treated with 5.5, 10, 20 or 30 mM d-glucose for 24 h were determined by western blot. **B**, **B1** The protein levels of ALPK1 in HK-2 cells treated with 30 mM d-glucose for different periods of time (0, 12, 24 or 48 h) were determined by western blot. Experiments were performed in triplicate. Data were presented as mean ± SD (*ns* no significance, **P < 0.01, ****P < 0.0001)

To investigate the effect of high glucose on tubular ALPK1 expression in HK-2 cells, we exposed cells to D-glucose at different concentrations (5.5, 10, 20, 30 mM) for 24 h (Fig. 4A, A1) and then to 30 mM d-glucose for different periods of time (0, 12, 24, 48 h) (Fig. 4B, B1). We found that the protein level of ALPK1 increased in a time- and concentration-dependent manner with high glucose treatment (Fig. 4).

### ALPK1 knockdown decreased the HG-induced pyroptosis and fibrosis in HK-2 cells

**Fig. 5:**
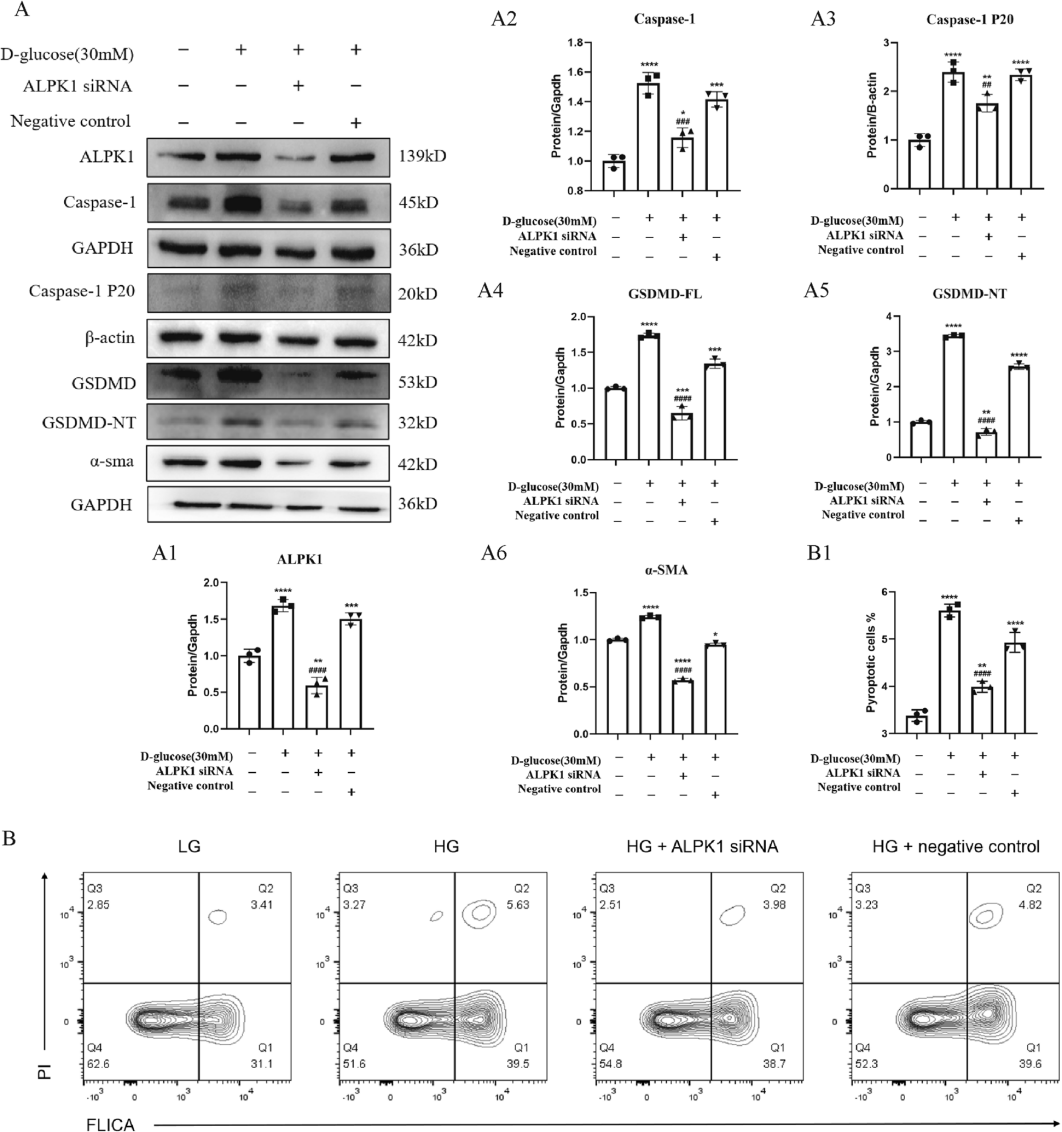
HK-2 cells were treated with ALPK1 siRNA or non-target siRNA for 6 h prior to 24 h-HG (30 mM) treatment. **A** Densitometry analysis shows knocking down ALPK1 using siRNA transfection downregulated ALPK1 (**A1**), caspase-1 (**A2**), caspase-1 P20 (**A3**), GSDMD (**A4**), GSDMD-NT (**A5**) and α-SMA (**A6**) which increased under high glucose environment. **B**, **B1** Flow cytometry analysis shows high glucose (5.63%) increased proportion of pyroptotic HK-2 cells compared with the control (3.41%) and the proportion was reversed after knockdown of ALPK1 (3.98%). Quadrant 2 presented ratio of pyroptosis cells. Experiments were performed in triplicate. Data were presented as mean ± SD. *P < 0.05, **P < 0.01, ***P < 0.001, ****P < 0.0001 (compared with LG group). ^#^*P* < 0.05, ^##^*P* < 0.01, ^###^*P* < 0.001, ^####^*P* < 0.0001 (compared with HG group)

To explore the role of ALPK1 in pyroptosis in vitro, the cleavage of caspase-1 and GSDMD were detected in HK-2 cells under HG condition with ALPK1 small interfering RNA (siRNA) transfection. The results showed that the expressions of ALPK1, caspase-1, caspase-1 P20, GSDMD and GSDMD-NT (Fig. 5A, A1–5) were increased in HK-2 cells exposed to HG condition, while decreased by pretreatment with ALPK1 siRNA. Similar results were also found in the expression of α-SMA (Fig. 5A, A6). Furthermore, the down-regulated percentage of pyroptotic cells (FLICA^+^ and PI^+^ cells in quadrant 2) was observed in HK-2 cells under HG ambience with ALPK1 siRNA transfection by flow cytometry analysis (Fig. 5B, B1).

### ALPK1 knockdown decreased the phosphorylation and nuclear translocation of NF-κB in HK-2 cells under HG condition

**Fig. 6:**
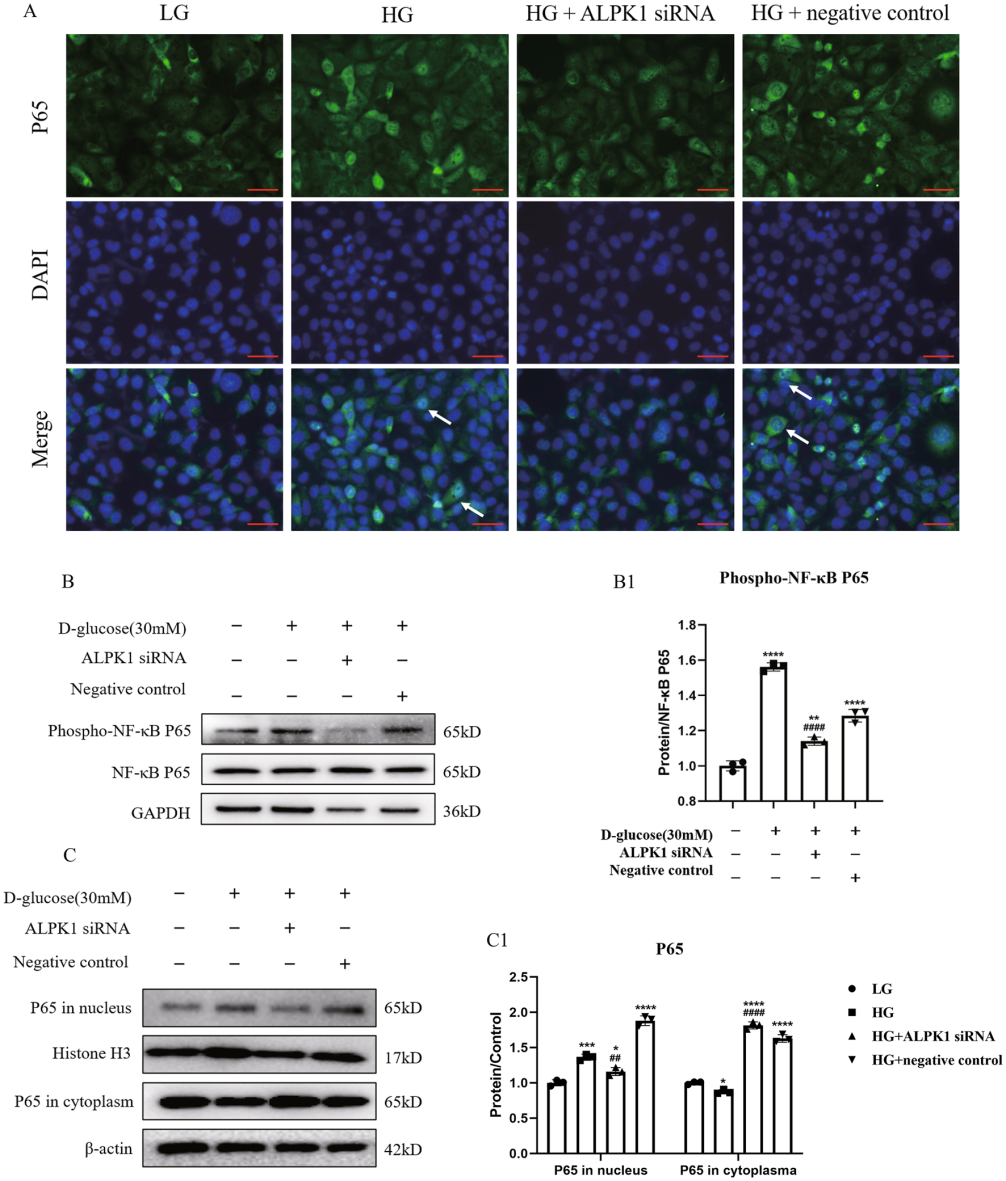
HK-2 cells were treated with ALPK1 siRNA or non-target siRNA for 6 h prior to 24 h-HG (30 mM) treatment. **A** Representative images of immunofluorescence staining for NF-κB P65 (green) with nuclei marked by DAPI (blue). ALPK1 knockdown inhibited the nuclear translocation of NF-κB P65 (arrow shows) (scale bar, 50 μm; magnification ×400). **B**, **B1** The protein level of phospho-NF-κB P65 in four groups were determined by western blot. **C**, **C1** Western blot determined cellular distribution of P65 in HK-2 cells. Experiments were performed in triplicate. Data were presented as mean ± SD. *P < 0.05, **P < 0.01, ***P < 0.001, ****P < 0.0001 (compared with LG group). ^#^*P* < 0.05, ^##^*P* < 0.01, ^###^*P* < 0.001, ^####^*P* < 0.0001 (compared with HG group)

IF staining and WB analysis were performed to verify the effect of ALPK1 on the NF-κB translocation and phosphorylation in HK-2 cells. As Fig. 6A showed, the nuclear translocation of NF-κB in HK-2 cells was increased under HG condition, while reversed by ALPK1 siRNA transfection. Meanwhile, the increased expression of phospho-NF-κB was down-regulated by the ALPK1 siRNA treatment (Fig. 6B, B1). Consistently, nuclear and cytoplasmic fractionation were carried out and NF-κB nuclear entry was also detected in HG group by Western blotting, which was inhibited by ALPK1 siRNA (Fig. 6C, C1). These results suggested that pyroptosis was regulated by ALPK1 through NF-κB translocation and phosphorylation in HK-2 cells under HG ambience.

### ALPK1 knockdown inhibited translocation of GSDMD to the cell membrane and suppressed release of IL-18

**Fig. 7:**
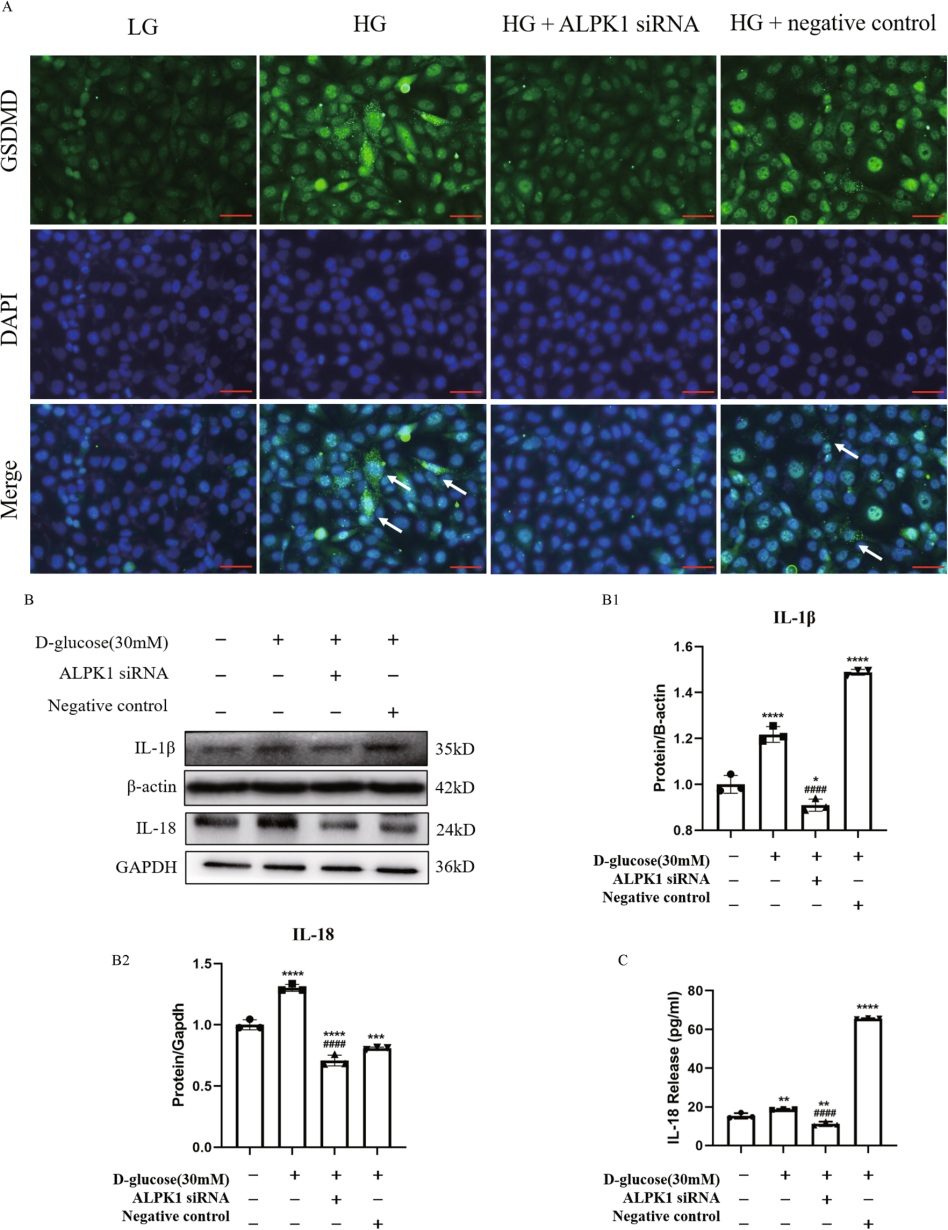
HK-2 cells were treated with ALPK1 siRNA or non-target siRNA for 6 h prior to 24 h-HG (30 mM) treatment. **A** Representative images of immunofluorescence staining for GSDMD (green) with nuclei marked by DAPI (blue) show knockdown of ALPK1 inhibited translocation of GSDMD to the cell membrane (arrow shows) induced by high glucose (scale bar, 50 μm; magnification ×400). **B** The protein levels of IL-1β and IL-18 in HG-treated HK-2 cells were determined by western blot. **C** IL-18 level in supernatant of HK-2 cells was measured by ELISA. Experiments were performed in triplicate. Data were presented as mean ± SD. *P < 0.05, **P < 0.01, ***P < 0.001, ****P < 0.0001 (compared with LG group). ^#^*P* < 0.05, ^##^*P* < 0.01, ^###^*P* < 0.001, ^####^*P* < 0.0001 (compared with HG group)

Active N-terminal fragments of GSDMD translocate to membrane and mediate the formation of cell membrane pores to induce pyroptosis and promote the following secretion of IL-1β and IL-18. As shown in Fig. 7A, intensity and membranous translocation of GSDMD were increased in HG environment, while ALPK1 siRNA alleviated activation of GSDMD. Western blot results revealed that the protein levels of IL-1β and IL-18 in cell lysates were also increased under HG treatment and these changes were suppressed by knockdown of ALPK1 expression using siRNA (Fig. 7B). Similarly, ELISA showed that IL-18 level in the supernatant was increased after HG treatment, while reversed by ALPK1 siRNA transfection. (Fig. 7C). These data suggested that ALPK1 activates pyroptosis pathway by regulating translocation of GSDMD to the cell membrane and release of inflammatory factors including IL-18 in HK-2 cells under HG condition.

## Discussions

It has been generally recognized that inflammatory mechanism is attributed to the development of tubular injury and renal fibrosis in DN. Previous studies showed ALPK1 is highly associated with inflammatory responses in chronic diseases such as chronic kidney disease (CKD) and type 2 DM [15–18]. ALPK1 is identified as a mediator of inflammatory cytokines CCL2 and CCL5, and as the upstream kinase to induce the activation of NF-κB and production of pro-inflammatory cytokine, including IL-1β and IL-8. Previous study has also proved that ALPK1 enhanced production of IL-1β in the kidney of experimental models of hyperglycemia and resulted in the induction of fibrotic renal injury [12, 19]. Considering the critical regulatory role of ALPK1 in inflammation under hyperglycemia conditions, it is reasonable to assume that ALPK1 participates in the development of renal injury in hyperglycemic condition. To study the molecules that participate in kidney damage in the diabetic kidney, we first performed bioinformatics analysis and identified that ALPK1 was differentially expressed in the kidneys of diabetic and control mice (Fig. 1). IHC staining confirmed that the expression of ALPK1 was significantly increased in renal tubules of DN patients, which was positively correlated with the chronic pathological index of interstitial fibrosis and tubular atrophy and renal function, accompanied by interstitial macrophage infiltration (Fig. 2). These results indicated that ALPK1 might be involved in the pathogenesis of renal tubular injury in DN patients. However, the mechanism of ALPK1 on tubular injury of DN still needs to be identified.

Previous researches have confirmed ALPK1 promotes renal fibrosis in mice by promoting the production of pro-inflammatory cytokines IL-1β, IL-8 and TNF-α [12]. In recent years, it has been proved that caspases protein family regulates pyroptosis by cutting GSDMD and then the N-terminal domain of GSDMD could release from the auto-inhibition of C-terminal domain [20], accompanied by the release of pro-inflammatory factors IL-1β and IL-18 [21, 22]. These studies suggest that ALPK1 may be involved in the regulation of cell damage through the classical pathway of cell pyroptosis. However, there is no relevant research on the mechanism of ALPK1 in DN. In this study, the heat map of DEGs and PPI network analysis show ALPK1, caspase-1 and GSDMD were up-regulated in the DN mice and ALPK1 is associated with pyroptosis-related proteins (Fig. 1). In vitro study, ALPK1 increased in time- and concentration-dependent manner in HK-2 cells by high glucose treatment (Fig. 4). Then we established a type 2 DN mouse model, and found that the expressions of ALPK1, pyroptosis-related protein caspase-1, GSDMD-NT and fibrosis-related protein α-SMA were significantly increased in DN mice (Fig. 3). In vitro, the down-regulation of ALPK1 expression in HK-2 cells reversed the up-regulation of pyroptosis-related protein expressions and the increase of pyroptosis cell ratio induced by high glucose. Down-regulated ALPK1 also decreased the renal fibrosis index α-SMA (Fig. 5). These results suggest that ALPK1 is involved in the process of renal tubular epithelial injury and renal fibrosis in DN by regulating the caspase-1-GSDMD related classical pyroptosis pathway.

The mechanism for activation of ALPK1 by high glucose in DN is not clear. In the development of tubular injury in DN, glucose was transferred into renal cells by facilitative transporters such as glucose transporter (GLUT)-1 and -4, and activated various cellular events and signaling pathways, including generation of advanced glycation end products (AGEs) and reactive oxygen species (ROS), activation of the PKC and JAK-STAT pathways [23]. ALPK1 was recently proved as a cytosolic PRR specific for ADP-β-d-manno-heptose (ADP-heptose) [8], which mediates immune responses to diverse bacterial pathogens. As a cytosolic receptor, ALPK1 might be activated by AGEs or inflammatory factors generated by high glucose. However, the specific mechanism of how high glucose activates the ALPK1-regulated pyroptosis pathway still needs to be investigated in the future.

Previous studies has proved that NF-κB signaling pathway was involved in pyroptosis in various diseases [24–26]. ALPK1 was recently reported to activate NF-κB and subsequently induce the production of pro-inflammatory cytokines and chemokines, which mediates innate immune responses and inflammatory injury [27, 28]. Previous studies on the pathogenesis of acute renal failure have demonstrated that NF-κB inhibitors can significantly reduce the production of IL-1β and IL-18 in albumin-stimulated HK-2 cells [29]. Our previous study has also proved that the activation of NF-κB is involved in pyroptosis pathway activation in renal tubular cells of DN [6]. These results indicated that the activation of NF-κB is required for ALPK1-mediated pyroptosis pathway. In this study, phospho-NF-κB P65 was significantly increased in DN mice and HK-2 cells (Figs. 3 and 6). IF staining and WB analysis showed that high glucose induced increased phosphorylation and nuclear translocation of NF-κB in HK-2 cells, which could be reversed by ALPK1 siRNA (Fig. 6). These results suggest that ALPK1 activates the phosphorylation of NF-κB, and the activated NF-κB is transferred from cytoplasm to nucleus to participate in the activation process of pyroptosis pathway. NF-κB is a downstream regulatory molecule of ALPK1 in tubular cells injury of DN.

In the process of cell pyroptosis, GSDMD-N terminal is cleaved by caspase-1 and mediates the formation of cell membrane pores by combination with phosphorylated phosphatidylinositol and other lipid components, resulting in the release of IL-1β, IL-18 and other inflammatory factors [30]. We observed the distribution changes of GSDMD in high glucose environment by IF staining in HK-2 cells. Results showed that the obvious aggregation of GSDMD in the membrane was increased in HK-2 cells under high glucose environment, and inhibition of ALPK1 could reverse above situation. These results suggest that the transfer of GSDMD from cytoplasm to cell membrane and pore-forming are the key links of ALPK1-regulated pyroptosis injury of tubular cells in high glucose ambience. Inhibition of ALPK1 reversed the increased protein expression and cell supernatant level of inflammatory factor IL-18 in HK-2 with high glucose treatment. Recent study confirms renal TEC-derived IL-1β could polarizes macrophages towards a proinflammatory phenotype, resulting in inflammatory injury of kidney in high glucose ambience [31]. Our study has also shown increased macrophages in renal interstitium of DN patients, which were negatively correlated with eGFR and positively correlated with IFTA scores (Fig. 2). These confirmed that ALPK1 is involved in the regulation of renal tubular epithelial cell pyroptosis in DN, which leads to the release of IL-1β and IL-18 and activation of macrophages, further aggravating tubular cell injury.

Our study has some limitations. The mechanism of how hyperglycemia activates ALPK1 and how ALPK1-NF-κB signal pathway activates GSDMD-related canonical pyroptosis are unclear. Transgenic mice with tubular-specific deletion of ALPK1 may contribute to the further study. We will further improve in future research.

## Conclusion

In conclusion, hyperglycemia activates ALPK1 in renal TECs, leading to phosphorylation of NF-κB, which could contribute to release of the N-terminal domain of GSDMD after cleavage by caspase-1. GSDMD-NT inserts into the cell membrane lipid bilayer, leading to pyroptosis-related tubular injury. The consequently release of IL-1β and IL-18 activates macrophages in renal interstitium and aggravates tubular injury and renal fibrosis.

## Supplementary Information

**Additional file 1: Table S1.** Clinical characteristics.

## Data Availability

Not applicable.

## Abbreviations

DN: Diabetic nephropathy
IHC: Immunohistochemistry
HG: High glucose
siRNA: Small interfering RNA
TECs: Tubular epithelial cells
GSDMD-NT: N-terminal of GSDMD
ALPK1: Alpha‐kinase 1
CKD: Chronic kidney disease
type 2 DM: Type 2 diabetes mellitus
DEGs: Differentially expressed genes
HK-2: Human renal proximal tubular epithelial cells
FBS: Fetal bovine serum
HFD: High-fat diet
STZ: Streptozotocin
T2D: Type 2 diabetic
HE: Hematoxylin–eosin
GML: Glomerular minor lesion
IF: Immunofluorescence
ELISA: Enzyme-linked immunosorbent assay
eGFR: Estimated glomerular filtration rate
IFTA: Interstitial fibrosis and tubular atrophy
PPI: Protein–protein interaction
