# Supplementary material for: Alpha‐kinase1 promotes tubular injury and interstitial inflammation in diabetic nephropathy by canonical pyroptosis pathway
Source: Biol Res. 2023 Feb 2;56:5. doi: 10.1186/s40659-023-00416-7 (PMC9893546; doi:10.1186/s40659-023-00416-7)
Supplement: Supplementary file 1 — Additional file 1: Table S1. Clinical characteristics. [file 40659_2023_416_MOESM1_ESM.docx]

**Table S1. Clinical characteristics.**

|  | GML(n=5) | DN(n=8) |
| --- | --- | --- |
| Age (yr) | 26.2±15.42 | 50.88±11.67 |
| Sex (male/female) | 2/3 | 6/2 |
| ALB (g/L) | 40.22±11.97 | 33.09±6.88 |
| Blood glucose (mmol/L) | 5.57±0.04 | 8.42±2.38 |
| Hb (g/L) | 164±11.31 | 107.14±19.95 |
| Scr (μmol/L) | 62.04±14.63 | 280.85±263.32 |
| BUN (mmol/L) | 4.77±1.36 | 9.83±6.45 |
| UA(μmol/L) | 260.64±85.18 | 364.94±138.64 |
| eGFR (mL/min per 1.73m^2^) | 123.78±13.98 | 48.02±31.38^a^ |
| Total cholesterol (mmol/L) | 4.98±1.95 | 5.34±0.87 |
| Triglyceride (mmol/L) | 1.39±0.81 | 2.58±1.16 |
| 24-h urine protein (g/d) | 768.53±582.97 | 5290±2032.14^b^ |
| UNAG (U/L) | 11.12±5.48 | 17.93±9.31 |

ALB, Levels of albumin; Hb, Hemoglobin; Scr, Serum creatine; BUN. Blood urea nitrogen; UA, Uric acid; eGFR, estimated glomerular filtration rate; UNAG, urinary N-acetyl-β-D-glycosaminidase, GML, glomerular minor lesion; DN, diabetic nephropathy. The data are presented as the means ± SDs.

^a^*p*＜0.001 versus GML group.

^b^*p*＜0.001 versus GML group.
